# Association between the TyGBS index and all-cause and cardiovascular mortality in diabetic stroke patients

**DOI:** 10.3389/fendo.2025.1551825

**Published:** 2025-09-30

**Authors:** Gaggui Lu, Xingyu Miao, Facai Meng, Kaifei Liu, Xiaomei Yang, Man Zhang

**Affiliations:** ^1^ Department of Neurosurgery, Shaanxi Provincial People’s Hospital, Xi’an, China; ^2^ Department of Neurosurgery Nursing, Shaanxi Provincial People’s Hospital, Xi’an, China; ^3^ Department of Gynecology, Shaanxi Provincial People’s Hospital, Xi’an, China

**Keywords:** TyGBS, diabetes, stroke, all-cause mortality, cardiovascular mortality, oxidative stress, insulin resistance

## Abstract

**Background:**

Stroke patients with diabetes face elevated mortality risks, posing a global public health challenge. The TyG (Triglyceride-Glucose) index and Oxidative Balance Score (OBS) are potential predictors of mortality, but their combined impact remains unclear. This study explores the association between Triglyceride-Glucose to Oxidative Balance Score Ratio (TyGBS) and all-cause and cardiovascular mortality in diabetic stroke patients.

**Methods:**

Data from 556 diabetic stroke patients in the NHANES 1999–2018 cohort were analyzed. Mortality outcomes were determined using the National Death Index. TyGBS, calculated as the TyG index divided by OBS, was assessed for its association with mortality through Kaplan-Meier analysis, multivariable Cox regression, restricted cubic splines (RCS), and subgroup analyses.

**Results:**

Over a mean follow-up of 73 months, 210 (49.4%) patients died, including 65 (15.3%) from cardiovascular causes. Higher TyGBS levels were significantly associated with increased mortality risks. Kaplan-Meier analysis showed the lowest mortality in the lowest TyGBS quartile (Q1) and the highest in Q4 (log-rank p<0.001). Cox regression revealed that each unit increase in TyGBS raised all-cause mortality risk by 360% (HR 4.60, 95% CI 3.21–6.59) and cardiovascular mortality risk by 357% (HR 4.57, 95% CI 2.43–8.60). The RCS analysis indicated a nonlinear association, showing a significant increase in mortality risk when TyGBS was less than 1.163 (p for nonlinearity = 0.002).

**Conclusion:**

TyGBS, a novel ratio integrating metabolic and oxidative pathways, demonstrates a critical clinical threshold for prioritizing interventions in diabetic stroke.

## Introduction

Diabetes is a global chronic metabolic disorder, with both its incidence and mortality rates showing a continuous upward trend (1–3). Diabetic patients face a significantly increased risk of cardiovascular complications, with stroke being one of the most severe complications and a primary cause of mortality in this population (4–6). Research indicates that the risk of stroke in diabetic patients is 2 to 4 times higher than in non-diabetic individuals, with a poorer prognosis (7). In the progression of diabetic stroke outcomes, insulin resistance and oxidative stress play pivotal roles, with a bidirectional relationship existing between them. On one hand, oxidative stress can induce insulin resistance by promoting inflammation and impairing insulin signaling pathways. For instance, elevated levels of ROS can activate serine kinases, inhibiting insulin receptor substrates (IRS) and disrupting the phosphoinositide 3-kinase (PI3K)/Akt signaling pathway crucial for glucose uptake (8, 9). On the other hand, insulin resistance can exacerbate metabolic dysfunction by enhancing fat breakdown and releasing free fatty acids, leading to increased oxidative stress (8, 10). Insulin resistance can lead to metabolic dysregulation and increased oxidative stress, which, in turn, exacerbates insulin resistance and promotes vascular complications. Accurately assessing these dual changes can offer valuable insights into the potential mortality risk of preventing stroke complications in diabetes.

High triglycerides play a dual pathological role in the progression of diabetic stroke. Firstly, the abnormally elevated levels of circulating free fatty acids (FFA) directly induce vascular endothelial inflammation, promoting monocyte adhesion and atherosclerotic plaque formation through the activation of signaling pathways such as the nuclear factor κB (11). Secondly, in the state of hypertriglyceridemia, the liver excessively synthesizes very low-density lipoprotein, leading to an elevated ratio of small, dense low-density lipoprotein. This subtype of lipoprotein is more prone to penetrating vascular endothelium and accumulating in the subendothelial space, thereby hastening the formation of lipid cores (12–14). The synergistic effect of elevated triglycerides and high blood glucose significantly activates the protein kinase C-beta pathway, leading to the overexpression of vascular endothelial growth factor and ultimately compromising the structural integrity of the blood-brain barrier (15, 16).

The glycerol-triglycerides-glucose index (TyG index) has been validated as a simple and reliable indicator in the assessment of insulin resistance, showing close associations with various metabolic disorders such as diabetes and cardiovascular diseases (17). The latest research reveals a U-shaped relationship between the TyG index and overall mortality as well as cardiovascular mortality in patients with diabetes (7). Lower TyG levels are significantly positively associated with a higher risk of long-term all-cause mortality in young populations with stroke and diabetes (18, 19). However, the TyG index still has limitations in predicting the risk of long-term cardiovascular complications in diabetic patients (20). In the assessment of oxidative stress, the Oxidative Balance Score (OBS) serves as a comprehensive indicator of the body’s oxidative-antioxidant status, encompassing various aspects such as diet and lifestyle (21). Higher levels of OBS are associated with a reduced risk of developing diabetes, according to research findings (22). It is also associated with a reduction in all-cause mortality and cardiovascular mortality in patients with diabetes (23). Although OBS serves as a new indicator for assessing the risk of mortality, it still has some limitations (24). Firstly, the lack of standardized calculation criteria leads to variations in research outcomes. Therefore, when assessing the risk of mortality using the OBS, it may be necessary to integrate other indicators for a comprehensive evaluation of an individual’s mortality risk. Based on the evidence presented, integrating the TyG index with OBS may offer a new perspective for assessing the risk of stroke-related mortality and prognosis in diabetic patients.

However, there have been no reports on the predictive ability of the TyG index in conjunction with OBS as a comprehensive indicator in diabetic patients with stroke. Considering the significant roles of insulin resistance and oxidative stress in the pathogenesis of stroke in diabetes, investigating the relationship between TyGBS and mortality in diabetic stroke holds crucial clinical relevance. This aids in early identification of high-risk individuals and provides a theoretical basis for developing personalized prevention and treatment strategies.

Based on the background provided, we have developed a novel index, the TyGBS index (Triglyceride-Glucose to Oxidative Balance Score Ratio). The study aims to investigate the association between TyGBS index and all-cause mortality as well as cardiovascular mortality in patients with diabetes and stroke by analyzing data from a national health and nutrition examination survey.

## Method

### Study population

The National Health and Nutrition Examination Survey (NHANES) is a nationally representative survey conducted by the National Center for Health Statistics (NCHS), which operates under the Centers for Disease Control and Prevention (CDC) in the United States. The NHANES research protocol is approved by the NCHS ethics review board, and all participants provide written informed consent.

**Figure 1:**
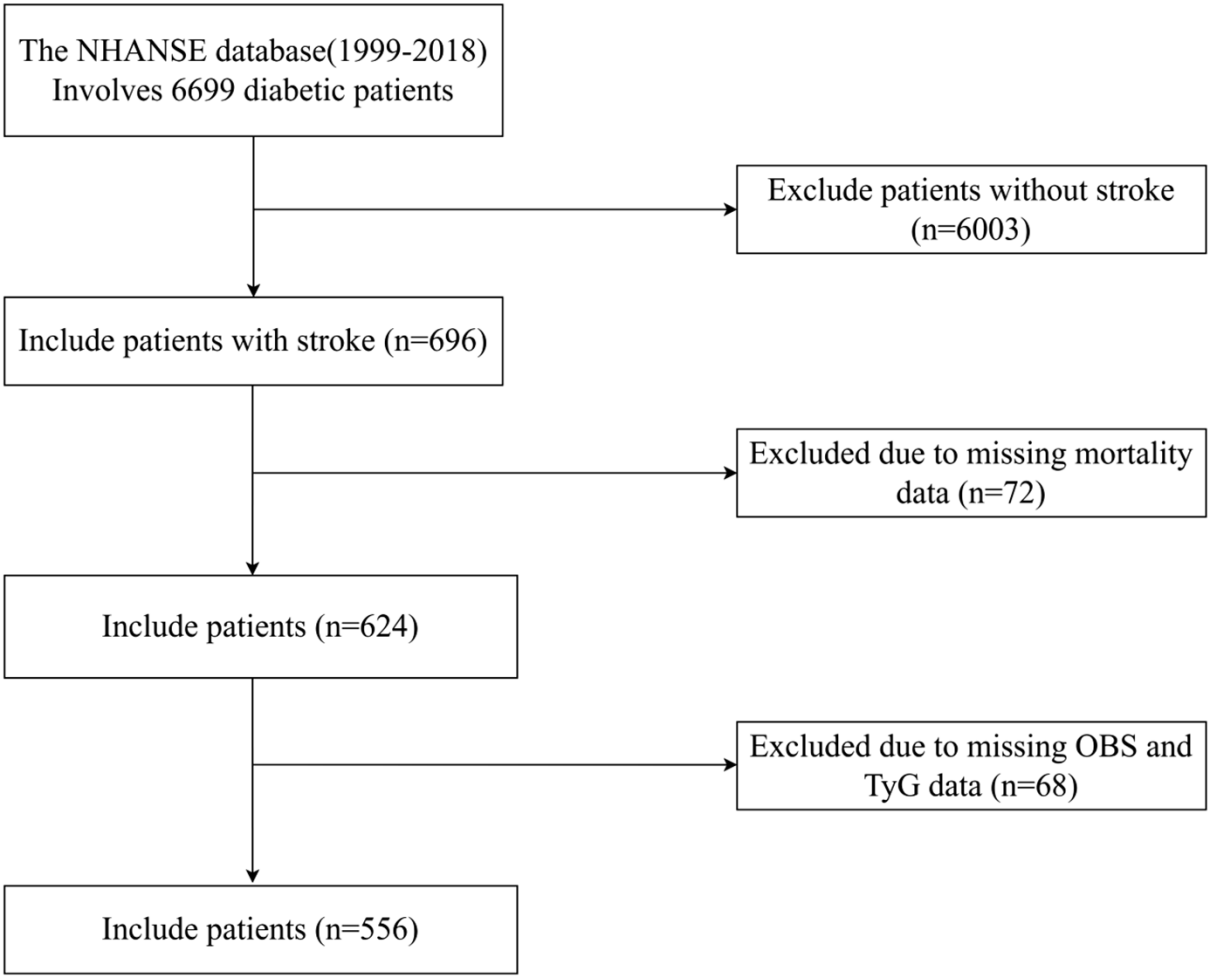
Flow chart of the study participant selection process.

The data used in this study were derived from the NHANES database, covering 10 cycles (1999-2000, 2001-2002, 2003-2004, 2005-2006, 2007-2008, 2009-2010, 2011-2012, 2013-2014, 2015-2016, and 2017-2018). Initially, a total of 696 participants were enrolled. Exclusions were implemented for individuals with insufficient dietary and lifestyle data required for calculating oxidative balance scores, as well as for those with missing TyG and mortality data (n = 140). Our analysis ultimately comprised 556 participants (**Figure 1**). NHANES data lacks systematic documentation of specific medication use (e.g., statins, antihypertensives, antidiabetic agents in terms of type/dosage/duration), severity of comorbidities (such as stages of chronic kidney disease, active cancer), and details of dietary patterns (such as adherence to the Mediterranean diet). Despite adjusting the models for other covariates, these unmeasured factors may result in residual confounding effects.

### Definition of diabetes and stroke

Diabetes is defined as an individual meeting any of the following criteria (24): (1) fasting blood glucose ≥7.0 mmol/L or 2-hour post-oral glucose tolerance test level ≥11.1 mmol/L; (2) random blood glucose ≥11.1 mmol/L; (3) glycated hemoglobin (HbA1c) ≥6.5%; (4) self-reported diabetes diagnosis (“doctor told you have diabetes”) or current use of insulin.

Stroke is defined as a self-reported stroke diagnosis (“Has a doctor or other health professional ever told you that you had a stroke?”). This self-reported diagnosis has been shown to have good consistency and has been widely used in epidemiological studies related to NHANES (25).

### Variable of exposure: TyGBS index

The calculation of the Oxidative Balance Score (OBS) involves 16 dietary nutrients and 4 lifestyle factors, comprising 15 antioxidants and 5 pro-oxidants (26). The 16 nutrients were derived from the first dietary recall interview, including dietary fiber, carotenoids (retinol equivalents, RE), riboflavin, niacin, vitamin B6, total folate, vitamin B12, vitamin C, vitamin E (α-tocopherol equivalents, ATE), calcium, magnesium, zinc, copper, selenium, total fat, and iron. The four lifestyle factors are physical activity, body mass index (BMI), alcohol consumption, and smoking, with smoking intensity measured by cotinine levels. The five pro-oxidant components include total fat, iron, BMI, alcohol consumption, and cotinine, while the remaining factors are classified as antioxidants.

Calculate the TyG index using the formula Ln [fasting triglycerides (mg/dL) × fasting glucose (mg/dL)/2] (27).

Alcohol consumption was categorized into three groups based on previous literature (24). The groups were defined as: non-drinkers, light to moderate drinkers (women: 0–15 g/day; men: 0–30 g/day), and heavy drinkers (women:≥15 g/day; men: ≥30 g/day). These groups were assigned scores of 2, 1, and 0. Other components are scored based on the gender-stratified tertiles. For antioxidant ingredients, the scores are 0, 1, and 2 for the lowest, middle, and highest tertiles, respectively. In contrast, the highest score for the pro-oxidative components was 0 points, while the lowest score was 2 points. By summing the scores of each component, the total Oxidative Balance Score (OBS) is calculated, ranging from 3 to 36. A higher score indicates greater exposure to antioxidants.

The TyGBS index is defined as the ratio of the TyG index to the OBS oxidative balance score.

### Determination of mortality rate

Data on mortality was retrieved from the National Death Index (NDI) database[https://www.cdc.gov/nchs/data-linkage/mortality-public.htm], managed by the Centers for Disease Control and Prevention (CDC) in the United States. The follow-up period was determined from the baseline interview date until the occurrence of death or December 31, 2019, the most recent update in the NDI database. Cardiovascular mortality was identified through the International Classification of Diseases, 10th Revision (ICD-10) codes I00-I09, I11, I13, and I20-I51.

### Covariates

In this study, the considered covariates include age, gender, race/ethnicity (Mexican American, other Hispanic, non-Hispanic white, non-Hispanic black, and other races), education level (less than high school, high school or equivalent, above high school), marital status (married, single, cohabiting), poverty to income ratio (≤1.0, 1.0-3.0, >3.0), smoking history (defined as having smoked at least 100 cigarettes in a lifetime), alcohol history (defined as consuming at least 12 alcoholic drinks per year), and history of hypertension.

### Statistical analysis

Statistical analysis can be conducted following the guidelines provided by the CDC, available at https://www.cdc.gov/nchs/data-linkage/mortality.htm. Baseline characteristics were presented using quartiles for OBS-S. Continuous variables were expressed as mean ± standard deviation (SD), while categorical variables were presented as percentages. The chi-square test was employed to calculate p-values for categorical variables, and the Kruskal-Wallis rank sum test was used for continuous variables. Three Cox regression models (Model 1, Model 2, Model 3) were developed to assess the relationship between various indices in diabetic stroke patients and all-cause mortality. Model 1 included gender, age, race/ethnicity, education level, marital status, poverty income ratio, smoking history, alcohol history, hypertension history, and OBS. Model 2 comprised gender, age, race/ethnicity, education level, marital status, poverty income ratio, smoking history, alcohol history, hypertension history, and TyG index. Model 3 consisted of gender, age, race/ethnicity, education level, marital status, poverty income ratio, smoking history, alcohol history, hypertension history, and TyGBS index. Utilize the log-rank test and Kaplan-Meier (K-M) survival analysis to investigate differences in survival probabilities. Three multivariable Cox regression models (Model 1, Model 2, Model 3) were developed to assess the relationship between the TyGBS index in diabetic stroke patients and the overall mortality rate and cardiovascular mortality rate. Model 1 was unadjusted for covariates, Model 2 was adjusted for gender, age, and race/ethnicity, and Model 3 was adjusted for gender, age, race/ethnicity, education level, marital status, poverty income ratio, smoking history, alcohol history, and hypertension history. To examine the proportional hazards (PH) assumption, the goodness of fit test using Schoenfeld residuals was employed.

The segmented model was applied using the R segmented package, which estimates breakpoints and fits a separate linear model for each segment defined by these breakpoints. The value of breakpoints was determined based on model fit and statistical tests. To assess the overall association between TyGBS and mortality, the standard Cox regression model was compared to the piecewise Cox regression model. The log-likelihood ratio test (LRT) was used to determine if the piecewise model provided a significantly better fit than the standard Cox regression model, with P < 0.05 indicating a statistically significant difference. Subgroup analysis and interaction testing were performed to examine the association between the TyGBS index and mortality rates across various subgroups, following adjustment for gender, age, and race/ethnicity in Model 2.

All statistical analyses were conducted using R version 4.3.3. A two-tailed p-value of less than 0.05 was considered statistically significant.

## Results

### Baseline characteristics of the participants

**Table 1** presents the baseline characteristics of the study subjects, categorized according to quartiles of TyGBS. The study involved a total of 556 patients with diabetes comorbid with stroke. The average age of participants is 67.9 years (± 10.3), with the majority being non-Hispanic white. Compared to participants in the lowest quartile (Q1) of TyGBS, those in the highest quartile (Q4) are more likely to be males, younger, non-Hispanic whites, have less than 9 years of education, unmarried, experiencing higher levels of poverty, with a history of hypertension, and engaging in higher levels of alcohol consumption and smoking. The TyG index is high while the OBS score is low. There were statistically significant differences among the TyGBS groups in terms of smoking status, history of hypertension, TyG index, and OBS score.

**Table 1:** Patient demographics and baseline characteristics.

Characteristic	TyGBS				p-value
	Q1, N = 138^1^	Q2, N = 137^1^	Q3, N = 141^1^	Q4, N = 140^1^	
Gender					0.135^2^
Male	67 (48.6%)	71 (51.8%)	71 (50.4%)	86 (61.4%)	
Female	71 (51.4%)	66 (48.2%)	70 (49.6%)	54 (38.6%)	
Age	71 (62, 78)	70 (63, 77)	67 (61, 76)	67 (61, 77)	0.058^3^
Race/ethnicity					0.513^2^
Mexican American	19 (13.8%)	23 (16.8%)	18 (12.8%)	21 (15.0%)	
Other Hispanic	12 (8.7%)	12 (8.8%)	6 (4.3%)	4 (2.9%)	
Non-Hispanic White	61 (44.2%)	55 (40.1%)	61 (43.3%)	62 (44.3%)	
Non-Hispanic Black	34 (24.6%)	38 (27.7%)	46 (32.6%)	46 (32.9%)	
Other Race	12 (8.7%)	9 (6.6%)	10 (7.1%)	7 (5.0%)	
Education					0.103^2^
Less Than 9th Grade	29 (21.0%)	24 (17.5%)	24 (17.0%)	39 (27.9%)	
9-11th Grade	23 (16.7%)	25 (18.2%)	26 (18.4%)	33 (23.6%)	
High School Grad	34 (24.6%)	35 (25.5%)	37 (26.2%)	30 (21.4%)	
Some College	30 (21.7%)	41 (29.9%)	41 (29.1%)	29 (20.7%)	
College Graduate	22 (15.9%)	12 (8.8%)	13 (9.2%)	9 (6.4%)	
Marital status					0.514^2^
Married	70 (50.7%)	75 (54.7%)	71 (50.4%)	62 (44.3%)	
Widowed	34 (24.6%)	34 (24.8%)	30 (21.3%)	30 (21.4%)	
Divorced	19 (13.8%)	15 (10.9%)	28 (19.9%)	23 (16.4%)	
Separated	5 (3.6%)	2 (1.5%)	3 (2.1%)	6 (4.3%)	
Never married	6 (4.3%)	7 (5.1%)	8 (5.7%)	11 (7.9%)	
Living with partner	2 (1.4%)	3 (2.2%)	1 (0.7%)	6 (4.3%)	
Not recorded	2 (1.4%)	1 (0.7%)	0 (0.0%)	2 (1.4%)	
Poverty to income ratio					0.356^2^
≤1.0	25 (18.1%)	36 (26.3%)	37 (26.2%)	39 (27.9%)	
1.0–3.0	76 (55.1%)	64 (46.7%)	65 (46.1%)	65 (46.4%)	
>3.0	27 (19.6%)	26 (19.0%)	26 (18.4%)	18 (12.9%)	
Not recorded	10 (7.2%)	11 (8.0%)	13 (9.2%)	18 (12.9%)	
Smoking					0.006^2^
Yes	69 (50.0%)	71 (51.8%)	85 (60.3%)	96 (68.6%)	
No	69 (50.0%)	66 (48.2%)	56 (39.7%)	44 (31.4%)	
Drinking					0.104^2^
Yes	82 (59.4%)	76 (55.5%)	95 (67.4%)	94 (67.1%)	
No	56 (40.6%)	61 (44.5%)	46 (32.6%)	46 (32.9%)	
Hypertension history					0.047^2^
Yes	115 (83.3%)	105 (76.6%)	125 (88.7%)	120 (85.7%)	
No	23 (16.7%)	32 (23.4%)	16 (11.3%)	20 (14.3%)	
TyG	8.89 (8.37, 9.31)	9.17 (8.59, 9.64)	9.31 (8.92, 10.00)	9.74 (9.23, 10.39)	<0.001^3^
OBS	12.50 (12.00, 14.00)	10.00 (10.00, 11.00)	9.00 (8.00, 9.00)	7.00 (6.00, 8.00)	<0.001^3^

^1^n (%); Median (IQR).

^2^Pearson's Chi-squared test.

^3^Kruskal-Wallis rank sum test.

### The relationship between OBS, TyG, and TyGBS with overall mortality rate

As shown in **Table 2**, this study constructed three different multivariate Cox regression models. In Model 1 (OBS), an increase in OBS is associated with a 19% decrease in the risk of all-cause mortality for each unit increase in OBS (HR 0.81, 95% CI 0.77-0.86, *p < 0.001*).In model 2 (TyG), for each unit increase in TyG, there is a significant 50% increase in the risk of all-cause mortality (HR 1.50, 95% CI 1.27-1.76, *p < 0.001*).In model 3 (TyGBS), there was a significant increase in the risk of all-cause mortality with each incremental rise in TyGBS (HR 4.81, 95% CI 3.29-7.02, *p < 0.001*).Comparing the performance of three Cox proportional hazards models in terms of fitting, prediction error, and model selection criteria, Model 3 (TyGBS) shows the best overall performance.

**Table 2:** Cox model analysis of overall mortality rates based on different independent variables.

Characteristic	HR^1^	95% CI^1^	p-value	Characteristic	HR^1^	95% CI^1^	p-value	Characteristic	HR^1^	95% CI^1^	p-value
Model 1				Model 2				Model 3			
Gender				Gender				Gender			
Male	—	—		Male	—	—		Male	—	—	
Female	0.73	0.53, 0.99	0.045	Female	0.71	0.52, 0.97	0.032	Female	0.77	0.56, 1.06	0.109
Age	1.06	1.04, 1.07	<0.001	Age	1.07	1.05, 1.09	<0.001	Age	1.06	1.04, 1.08	<0.001
Race/ethnicity				Race/ethnicity				Race/ethnicity			
Mexican American	—	—		Mexican American	—	—		Mexican American	—	—	
Other Hispanic	0.94	0.49, 1.80	0.843	Other Hispanic	0.84	0.44, 1.61	0.597	Other Hispanic	1.04	0.54, 2.01	0.899
Non-Hispanic White	1.08	0.73, 1.61	0.699	Non-Hispanic White	1.13	0.76, 1.69	0.533	Non-Hispanic White	1.11	0.74, 1.65	0.611
Non-Hispanic Black	0.92	0.61, 1.38	0.679	Non-Hispanic Black	1.23	0.82, 1.84	0.314	Non-Hispanic Black	0.99	0.66, 1.49	0.971
Other Race	0.54	0.25, 1.17	0.119	Other Race	0.62	0.29, 1.35	0.227	Other Race	0.57	0.26, 1.24	0.154
Education				Education				Education			
Less Than 9th Grade	—	—		Less Than 9th Grade	—	—		Less Than 9th Grade	—	—	
9-11th Grade	1.14	0.79, 1.66	0.477	9-11th Grade	1.17	0.81, 1.71	0.404	9-11th Grade	1.20	0.82, 1.74	0.343
High School Grad	0.91	0.62, 1.33	0.630	High School Grad	0.82	0.56, 1.19	0.293	High School Grad	0.93	0.63, 1.36	0.709
Some College	1.00	0.67, 1.48	0.993	Some College	0.92	0.63, 1.35	0.682	Some College	1.05	0.71, 1.56	0.805
College Graduate	1.06	0.62, 1.82	0.830	College Graduate	1.00	0.58, 1.72	0.992	College Graduate	1.08	0.63, 1.87	0.770
Marital status				Marital status				Marital status			
Married	—	—		Married	—	—		Married	—	—	
Widowed	1.22	0.88, 1.68	0.232	Widowed	1.17	0.85, 1.61	0.345	Widowed	1.22	0.88, 1.68	0.237
Divorced	1.06	0.71, 1.58	0.771	Divorced	1.26	0.86, 1.86	0.241	Divorced	1.02	0.69, 1.52	0.919
Separated	1.67	0.71, 3.91	0.238	Separated	0.78	0.31, 1.93	0.586	Separated	1.23	0.52, 2.90	0.638
Never married	0.83	0.42, 1.66	0.599	Never married	0.78	0.39, 1.55	0.477	Never married	0.82	0.41, 1.63	0.573
Living with partner	1.27	0.45, 3.58	0.646	Living with partner	1.24	0.45, 3.45	0.676	Living with partner	1.14	0.41, 3.20	0.803
Not recorded	1.24	0.44, 3.47	0.688	Not recorded	0.76	0.27, 2.18	0.614	Not recorded	1.24	0.44, 3.49	0.689
Poverty to income ratio				Poverty to income ratio				Poverty to income ratio			
≤1.0	—	—		≤1.0	—	—		≤1.0	—	—	
1.0–3.0	1.25	0.91, 1.73	0.173	1.0–3.0	1.23	0.89, 1.70	0.214	1.0–3.0	1.33	0.96, 1.84	0.087
>3.0	0.92	0.58, 1.46	0.716	>3.0	0.82	0.52, 1.30	0.399	>3.0	0.95	0.59, 1.51	0.814
Not recorded	1.26	0.80, 1.97	0.323	Not recorded	1.13	0.72, 1.78	0.601	Not recorded	1.34	0.85, 2.11	0.210
Smoking				Smoking				Smoking			
Yes	—	—		Yes	—	—		Yes	—	—	
No	0.96	0.73, 1.27	0.798	No	0.90	0.68, 1.19	0.445	No	0.94	0.71, 1.23	0.637
Drinking				Drinking				Drinking			
Yes	—	—		Yes	—	—		Yes	—	—	
No	1.10	0.82, 1.49	0.525	No	0.92	0.68, 1.26	0.609	No	1.02	0.76, 1.39	0.878
OBS	0.81	0.77, 0.86	<0.001	TyG	1.50	1.27, 1.76	<0.001	TyGBS	4.81	3.29, 7.02	<0.001

^1^HR, Hazard Ratio; CI, Confidence Interval.

### The relationship between TyGBS and the overall mortality rate and cardiovascular mortality rate

**Figure 2:**
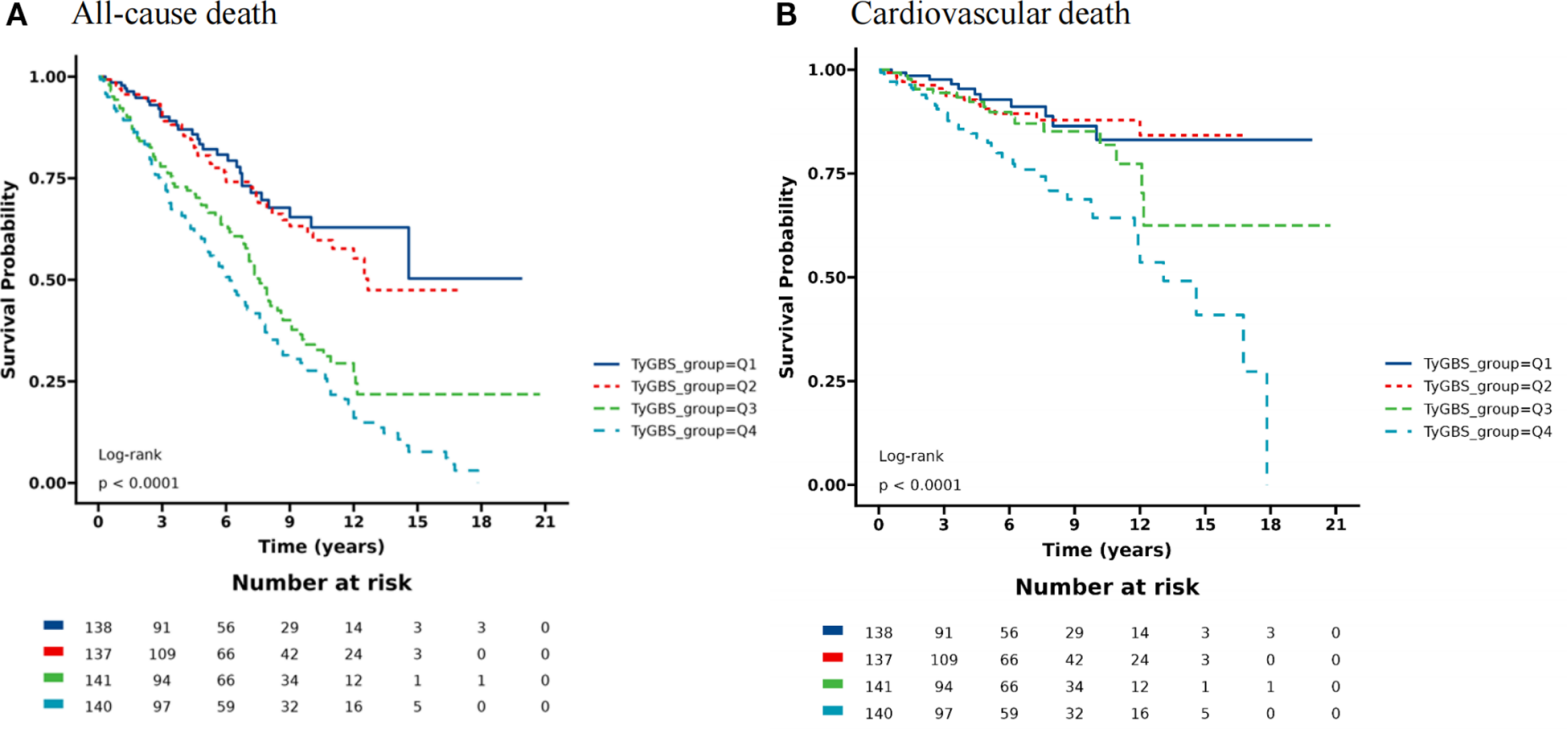
Kaplan-Meier curves for all-cause mortality **(A)** and cardiovascular mortality **(B)** among diabetic stroke patients stratified by quartiles (Q1-Q4) of TyGBS.

During a mean follow-up period of 73 months, out of 556 diabetic patients with a history of stroke, 210 individuals (49.4%) died, with 65 (15.3%) of them succumbing to cardiovascular diseases. The Kaplan-Meier (K-M) survival analysis revealed significant differences in overall and cardiovascular mortality rates among the four groups. The participants with the highest fourth quartile (Q4) of TyGBS had the highest mortality rate, while those with the lowest first quartile (Q1) had the lowest mortality rate (log-rank *p < 0.001*). The additional details of the K-M curve are illustrated in **Figure 2**.

**Table 3** shows the three Cox regression models that demonstrate the association between the TyGBS index and the overall mortality rate. In the unadjusted covariate model (Model 1), we observed a significant increase in the risk of all-cause mortality with an increase in the TyG index (HR 4.41, 95% CI 3.11-6.25, *p < 0.001*).Compared to the lowest quartile (Q1) of TyGBS, the highest quartile (Q4) is associated with a 227% increase in all-cause mortality risk (HR 3.27, 95% CI 2.20-4.85, *p < 0.001*).After adjusting for gender, age, and race in Model 2, for each unit increase in TyGBS, the risk of all-cause mortality increases by 360% (HR 4.60, 95% CI 3.21-6.59, *p < 0.001*).In this model, participants in the Q4 group of the TyGBS index had a 257% higher risk of all-cause mortality compared to the Q1 group (HR 3.57, 95% CI 2.40-5.31, *p <0.001*).In the fully adjusted model (Model 3), for every one unit increase in the TyGBS index, the overall risk of death increases by 388% (HR 4.88, 95% CI 3.33-7.14, *p<0.001*).When comparing Q4 to Q1, there was a 265% increase in the risk of mortality (HR 3.65, 95% CI 2.43-5.49, *p < 0.001*).This study compared the goodness-of-fit, predictive accuracy, and model selection performance of three Cox proportional hazards models for all-cause mortality and cardiovascular mortality outcomes. The results indicate that Model 2 performed the best overall, while Model 3 excelled in the Nagelkerke R-squared metric.

**Table 3:** Association between TyGBS and survival (Cox regression).

Characteristic	Model 1			Model 2			Model 3		
	HR^1^	95% CI^1^	p-value	HR^1^	95% CI^1^	p-value	HR^1^	95% CI^1^	p-value
All-cause death									
TyGBS	4.41	3.11, 6.25	<0.001	4.60	3.21, 6.59	<0.001	4.88	3.33, 7.14	<0.001
TyGBS (Quartile)									
Q1	—	—		—	—		—	—	
Q2	1.17	0.74, 1.84	0.497	1.23	0.78, 1.94	0.374	1.26	0.79, 2.01	0.333
Q3	2.43	1.61, 3.66	<0.001	2.75	1.82, 4.16	<0.001	2.91	1.90, 4.45	<0.001
Q4	3.27	2.20, 4.85	<0.001	3.57	2.40, 5.31	<0.001	3.65	2.43, 5.49	<0.001
P for trend			<0.001			<0.001			<0.001
Cardiovascular death									
TyGBS	4.88	2.62, 9.09	<0.001	4.57	2.43, 8.60	<0.001	4.97	2.57, 9.62	<0.001
TyGBS (Quartile)									
Q1	—	—		—	—		—	—	
Q2	1.19	0.54, 2.59	0.669	1.31	0.60, 2.87	0.502	1.25	0.55, 2.82	0.593
Q3	1.69	0.80, 3.59	0.172	1.90	0.89, 4.04	0.096	2.07	0.96, 4.49	0.064
Q4	3.30	1.68, 6.49	<0.001	3.44	1.74, 6.79	<0.001	3.73	1.86, 7.50	<0.001
P for trend			<0.001			<0.001			<0.001

^1^HR, Hazard Ratio; CI, Confidence Interval.

Model 1: no covariates were adjusted.

Model 2: adjusted for Gender, Age, and Race/ethnicity.

Model 3: adjusted for Gender, Age, Race/ethnicity, Education, Marital status, Poverty to income ratio, Smoking, Drinking, and Hypertension history.

**Figure 3:**
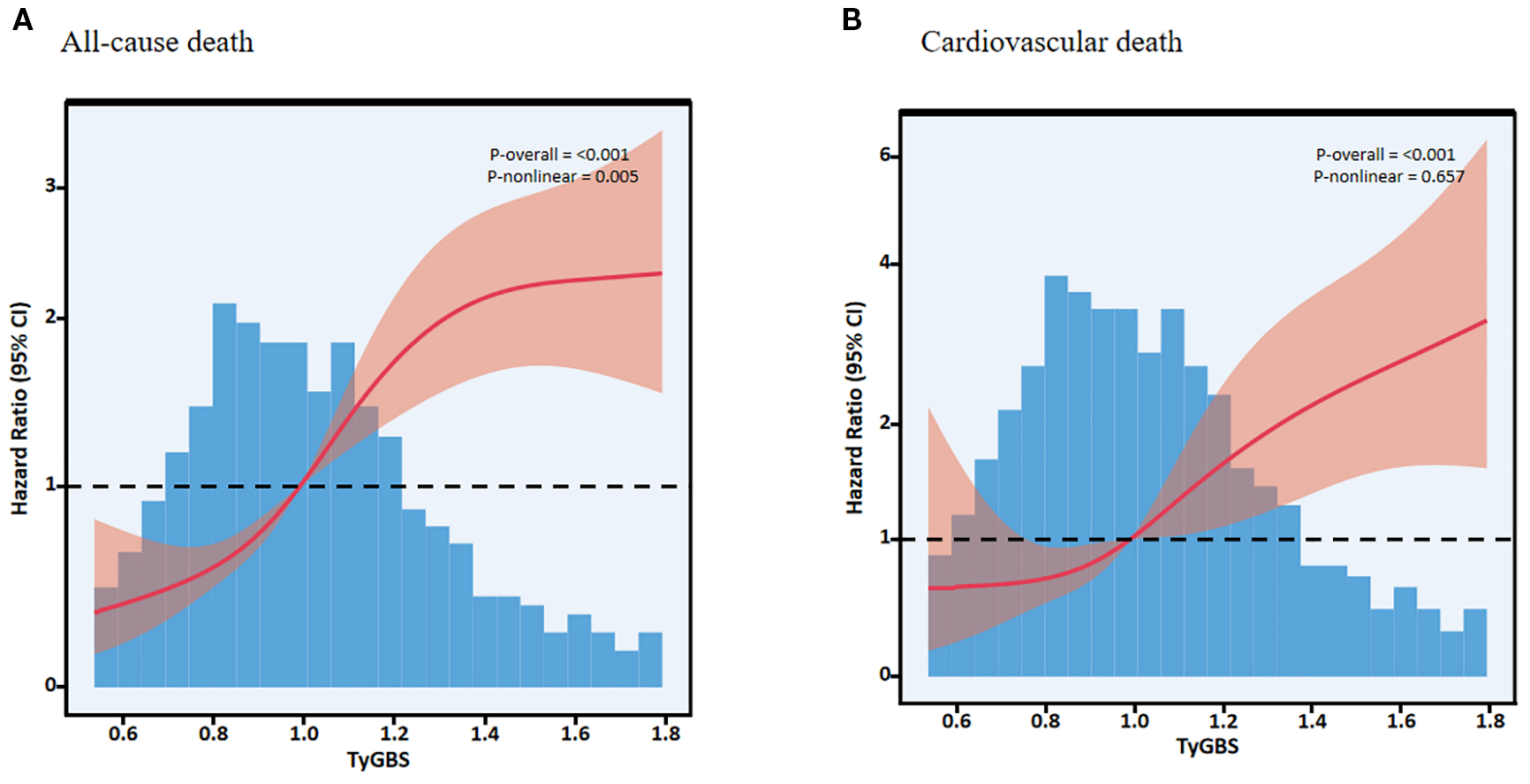
Restricted cubic spline curves showing the association of TyGBS with all-cause mortality **(A)** and cause-specific cardiovascular mortality **(B)** in diabetic stroke patients.

There is a curvilinear relationship between TyGBS and all-cause mortality, while there is no curvilinear relationship with cardiovascular mortality (**Figure 3**). The standard Cox regression analysis revealed a significant association between TyGBS and all-cause mortality (**Table 4**), with an adjusted hazard ratio of 4.59 (95% CI: 3.19-6.62, p<0.001). Piecewise regression identified a threshold effect at TyGBS=1.163. Below this threshold, TyGBS showed a strong positive association with mortality (adjusted HR = 20.18, 95% CI: 6.44-63.24, p<0.001), while no significant association was observed above the threshold (adjusted HR = 1.67, 95% CI: 0.71-3.92, p=0.239). The likelihood ratio test confirmed the superiority of the piecewise model over the standard Cox model (p=0.002), supporting the existence of a threshold effect.

**Table 4:** Threshold effect analysis of TyGBS on all-cause mortality.

	adjusted HR (95% CI)*	P-value
Fitting by standard Cox regression model	4.59 (3.19, 6.62)	<0.001
Fitting by piecewise Cox regression model (Break-Point = 1.163)		
TyGBS1 < 1.163	20.18 (6.44, 63.24)	<0.001
TyGBS1 ≥ 1.163	1.67 (0.71, 3.92)	0.239
Log likelihood ratio		0.002

*Adjusted for: Gender, Age, Race.ethnicity, Education.

### Subgroup analysis

**Figure 4:**
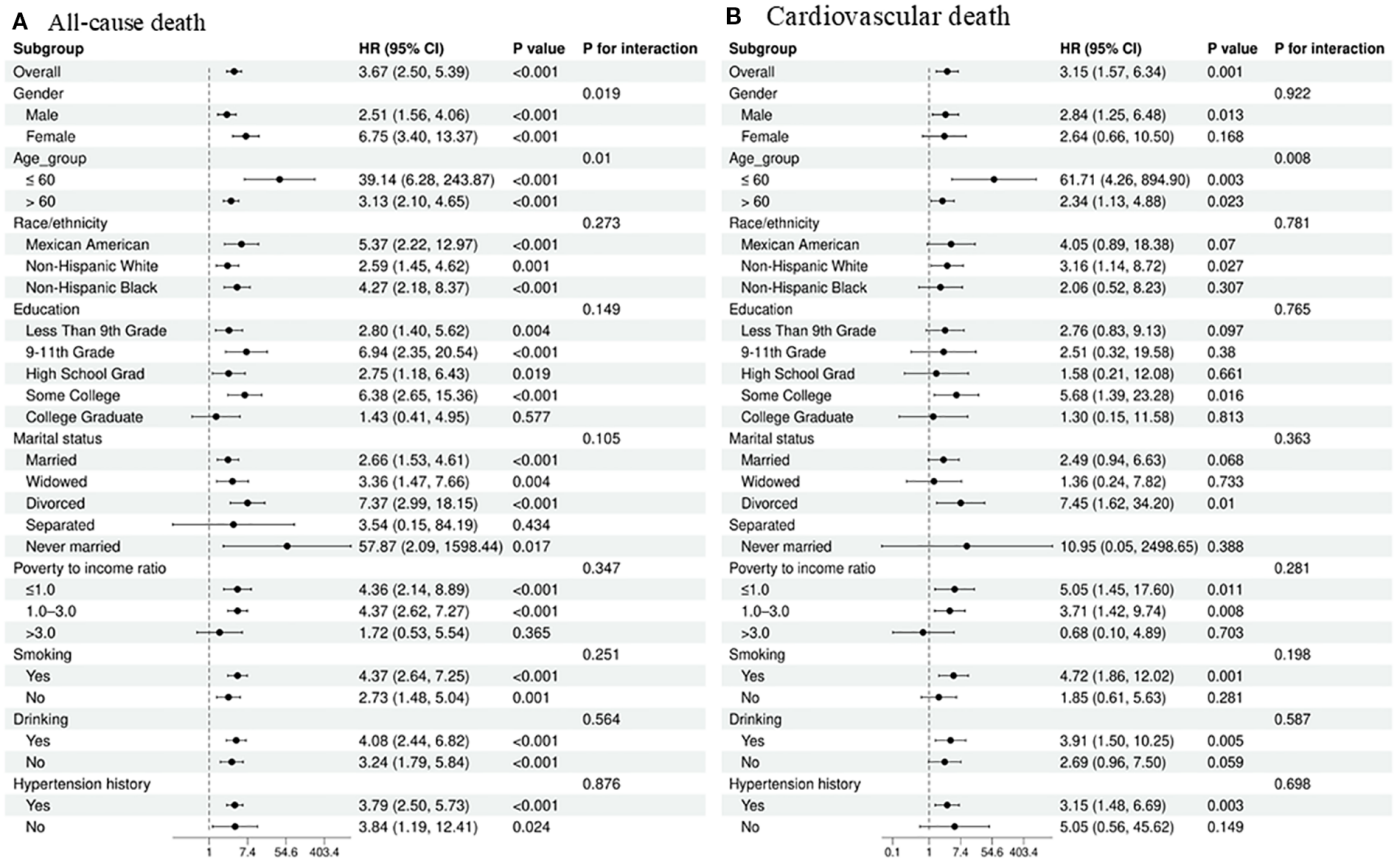
Subgroup analysis of the association between the TyGBS index and mortality outcomes in diabetic stroke patients: **(A)** all-cause mortality and **(B)** cardiovascular mortality.

This study employs a stratified analysis approach to assess the robustness of the regression results regarding the relationship between the TyGBS index and mortality rates among diabetic stroke patients in different subgroups. As shown in **Figure 4**, the results reveal a positive correlation between the TyGBS index and mortality rates in most subgroups, with statistical significance. Furthermore, gender and age appear to interact with the TyGBS index, suggesting a potential moderating effect *(p < 0.05*).

## Discussion

In this study, we assessed the correlation between the novel index TyGBS (the ratio between TyG and OBS) and the risk of mortality from stroke in individuals with diabetes. The research findings indicate a significant association between high TyGBS levels and increased overall mortality and cardiovascular disease mortality in individuals with diabetes and stroke. Our research findings offer a novel perspective on the dynamic assessment of mortality risk in individuals with diabetes and stroke.

Previous research has suggested a positive link between TyG and diabetes, as well as mortality due to stroke, whereas OBS has shown an inverse relationship with diabetes-related mortality. Nonetheless, the connection between TyG, OBS, and mortality in individuals with both diabetes and stroke remains uncertain (19, 24). This study reveals a positive correlation between TyG and mortality in diabetic patients with stroke, while OBS shows a negative correlation. This presents new evidence of the role of oxidative stress in assessing the risk of death in diabetic patients with stroke. Additionally, Xu et al. observed a significant negative correlation between an antioxidant-rich diet and TyG (28). A meta-analysis indicates that moderate intake of antioxidants can help reduce insulin resistance (29, 30). These findings suggest that the combination of low OBS and high TyG levels may exacerbate the risk of death from diabetes and stroke through a dual burden of oxidative stress and insulin resistance.

It is worth emphasizing that lipid-derived indicators such as TyG play a crucial role in cardiovascular risk assessment during the prediabetes stage (31). Therefore, identifying individuals at high cardiovascular risk at this stage is essential for early management. In this context, low-cost, non-invasive biomarkers such as the TyG index and TG/HDL-C ratio demonstrate significant potential. A recent study has confirmed a significant association between an elevated TyG index and the TG/HDL-C ratio and an increased cardiovascular risk in individuals with prediabetes or insulin resistance (32). This indicates their potential as effective tools for stratifying and managing cardiovascular risk in such populations. This discovery is consistent with our research findings.

Based on the findings of this study, our comparative analysis demonstrates that the Cox model constructed based on TyGBS outperforms models using TyG or OBS alone significantly in terms of goodness of fit, model complexity, and predictive accuracy. Therefore, we conclude that TyGBS serves as a novel and effective indicator for a more precise assessment of the mortality risk in diabetic patients with stroke. In the fields of diabetes and stroke, there is a relative scarcity of systematic evaluations on oxidative stress. Therefore, this study addresses this knowledge gap.

The mechanism underlying the association between TyGBS and mortality risk in stroke patients with diabetes may involve complex interactions among insulin resistance, oxidative stress, and inflammation. These factors exacerbate each other, collectively promoting the acceleration of atherosclerosis, endothelial dysfunction, and thrombus formation, ultimately leading to a higher mortality rate. Possible involvement of complex interactions among insulin resistance, oxidative stress, and inflammation. In diabetic patients, insulin resistance not only affects pancreatic insulin secretion but also leads to systemic metabolic disturbances, with oxidative stress playing a crucial role in this process (33, 34). The sustained elevation of oxidative stress levels may disrupt insulin signaling by downregulating key molecules, such as AMP-activated protein kinase (AMPK) and insulin receptor substrate (IRS) proteins (35, 36). This leads to a vicious cycle: Oxidative stress not only exacerbates insulin resistance but may also cause more severe oxidative damage to neurons during stroke events. Furthermore, oxidative stress interacts with inflammatory responses, further exacerbating the state of insulin resistance (37, 38). Therefore, future research should delve into the relationship between oxidative stress and insulin resistance to aid in the development of novel therapeutic strategies for enhancing the metabolic health of diabetic patients.

In our study, we found a nonlinear relationship between TyGBS and all-cause mortality, whereas a linear relationship was observed with cardiovascular mortality. The mortality rate sharply increases for TyGBS within the interval of less than 1.163. This finding contrasts with prior research. Liu et al. identified a U-shaped relationship between TyG in diabetic patients and the incidence of diabetes complications (39). Ni et al. demonstrated a linear correlation between OBS and all-cause mortality in patients with type 2 diabetes (40). These differences may stem from the varying regulatory effects of the TyG index across different age groups, as well as the relatively minor impact of OBS on mortality rates. Furthermore, changes in dietary habits and levels of physical activity in the population also significantly influence the interrelationship between TyG and OBS. In low- and middle-income countries, disparities in early-life environments may exacerbate susceptibility to metabolic disorders such as insulin resistance due to mismatches in unhealthy lifestyle choices in adulthood (41). After controlling for dietary and physical activity factors, this non-linear relationship may not be as significant in specific outcomes. The mortality rate sharply increases for TyGBS within the interval of less than 1.163.

In the sub-analysis, TyGBS was significantly associated with an increased risk of all-cause and cardiovascular mortality in individuals under 60 years of age. The increase in insulin resistance may be associated with the prolonged duration of illness in younger patients, potentially leading to exacerbated metabolic disturbances and oxidative stress, thereby triggering more severe complications (42). Furthermore, younger patients may experience increased physiological and psychological stress when dealing with chronic illnesses, further exacerbating the complexity of their condition. In individuals at high risk for stroke and with diabetes, the mortality rate significantly rises for TyGBS within an interval of less than 1.163. This finding underscores the importance of monitoring TyGBS levels in young diabetic patients in clinical practice.

### Significance of the study

Based on the findings above, the TyGBS index can serve as an effective measurement tool for identifying high-risk individuals for diabetes, stroke, and cardiovascular mortality. The mortality rate sharply increases for TyGBS within the interval of less than 1.163. Furthermore, since the TyGBS index requires the calculation of both TyG and OBS scores, it may be better suited as a long-term follow-up tool in clinical practice, especially among low to middle-income populations. Therefore, to achieve precise risk stratification for all-cause mortality patients, identifying individuals with oxidative stress disorders and insulin resistance requiring medical intervention, and targeting these individuals to improve their survival and cardiovascular health in a tailored manner, promoting the application of the TyG index in the population may be a beneficial and cost-effective approach.

### Strengths and limitations

In the analysis conducted, the absence of inflammatory biomarker data, such as C-reactive protein, in the National Health and Nutrition Examination Survey (NHANES) was noted. Consequently, the study did not incorporate this information. Moreover, the NHANES database did not provide specific data to differentiate between ischemic and hemorrhagic strokes, as well as between type 1 and type 2 diabetes. Therefore, the findings cannot be definitively extrapolated to distinguish between ischemic and hemorrhagic strokes. Given potential racial, dietary, and healthcare accessibility differences, the investigation was based on NHANES data, which is specific to the American population, thus limiting the generalizability of the results to other demographics. The study also faces potential residual confounding factors: the use of statins may concurrently reduce the TyG index and oxidative stress levels, potentially underestimating the true association strength between TyGBS and mortality (as users may exhibit both high TyGBS and low mortality risk); the precise measurement of the type, efficacy, and treatment adherence of antihypertensive drugs (such as ACEI/ARB) and glucose-lowering medications (such as SGLT2i/GLP-1RA) was not achieved; staging of chronic kidney disease (CKD) and active cancer was not fully stratified, potentially attributing part of the association between high TyGBS and mortality to these independent risk factors; although OBS scores encompass dietary antioxidant/pro-oxidant nutrients, unquantified dietary patterns (such as adherence to the Mediterranean diet) still present potential confounding. Future research should integrate medication history, comorbidity details, and dietary assessments to more accurately elucidate the prognostic value of TyGBS.

Although this study has certain limitations, it integrates lipid-glucose metabolism (TyG index) and oxidative stress (OBS score) pathways to establish the TyGBS composite index, overcoming the limitations of single-dimensional biomarkers. By accurately identifying the critical value of TyGBS through segmented regression models, this turning point can directly identify the subpopulation at high risk for diabetes and stroke in the brain, prioritizing metabolic-antioxidant combined interventions and providing a basis for precise stratified management.

## Conclusion

In the NHANES cohort (1999-2018), a non-linear association was observed between TyGBS and all-cause mortality: a significant increase in mortality risk was evident when TyGBS levels were below 1.163. This finding confirms the utility of TyGBS as a screening tool to identify diabetes stroke patients who would benefit from prioritized metabolic-oxidative comprehensive management.

## Data Availability

Publicly available datasets were analyzed in this study. This data can be found here: https://www.cdc.gov/nchs/nhanes/.

## References

[1] GBD 2021 Diabetes Collaborators. Global, regional, and national burden of diabetes from 1990 to 2021, with projections of prevalence to 2050: a systematic analysis for the Global Burden of Disease Study 2021. Lancet Lond Engl. (2023) 402:203–34. doi: 10.1016/S0140-6736(23)01301-6, PMID: 37356446 PMC10364581

[2] Hossain MJ Al-Mamun M Islam MR. Diabetes mellitus, the fastest growing global public health concern: Early detection should be focused. Health Sci Rep. (2024) 7:e2004. doi: 10.1002/hsr2.2004, PMID: 38524769 PMC10958528

[3] Abel Dale E Gloyn AL, Evans-Molina C Joseph JJ Misra S Pajvani UB. Diabetes mellitus-Progress and opportunities in the evolving epidemic. Cell. (2024) 187:2–3. doi: 10.1016/j.cell.2024.06.029, PMID: 39059357 PMC11299851

[4] Cho K-H Kwon SU Lee JS Yu S Cho A-H. Newly diagnosed diabetes has high risk for cardiovascular outcome in ischemic stroke patients. Sci Rep. (2021) 11:12929. doi: 10.1038/s41598-021-92349-y, PMID: 34155277 PMC8217241

[5] Vieira IH Carvalho TS Saraiva J Gomes L Paiva I. Diabetes and stroke: impact of novel therapies for the treatment of type 2 diabetes mellitus. Biomedicines. (2024) 12:1102. doi: 10.3390/biomedicines12051102, PMID: 38791064 PMC11117787

[6] Dardano A Bianchi C Garofolo M Del Prato S. The current landscape for diabetes treatment: Preventing diabetes-associated CV risk. Atherosclerosis. (2024) 394:117560. doi: 10.1016/j.atherosclerosis.2024.117560, PMID: 38688748

[7] Feng X Deng Y Chen C Liu X Huang Y Feng Y. Predictive value of triglyceride-glucose index for all-cause and cardiovascular mortality in patients with diabetes mellitus: A retrospective study. Int J Endocrinol. (2024) 2024:6417205. doi: 10.1155/2024/6417205, PMID: 39479579 PMC11524704

[8] Vargas-Vargas MA Saavedra-Molina A Gómez-Barroso M Peña-Montes D Cortés-Rojo C Miguel H. Dietary iron restriction improves muscle function, dyslipidemia, and decreased muscle oxidative stress in streptozotocin-induced diabetic rats. Antioxidants. (2022) 11:731. doi: 10.3390/antiox11040731, PMID: 35453417 PMC9030937

[9] Pieri BLDS Rodrigues MS Farias HR Silveira GDB Ribeiro VDSGDC Silveira PCL. Role of oxidative stress on insulin resistance in diet-induced obesity mice. Int J Mol Sci. (2023) 24:12088. doi: 10.3390/ijms241512088, PMID: 37569463 PMC10419159

[10] Mastrototaro L Roden M. Insulin resistance and insulin sensitizing agents. Metabolism. (2021) 125:154892. doi: 10.1016/j.metabol.2021.154892, PMID: 34563556

[11] Salsinha AS Socodato R Rodrigues A Vale-Silva R Relvas JB Pintado M. Potential of omega-3 and conjugated fatty acids to control microglia inflammatory imbalance elicited by obesogenic nutrients. Biochim Biophys Acta BBA - Mol Cell Biol Lipids. (2023) 1868:159331. doi: 10.1016/j.bbalip.2023.159331, PMID: 37172801

[12] Zhang M Yang D Wang J Wang D Xu J Wang Y. Association of serum lipidomic profiles with risk of intracranial aneurysm: A Mendelian randomization study. J Neurochem. (2025) 169:e16247. doi: 10.1111/jnc.16247, PMID: 39449543

[13] Sheng Y Gao F Zhu Z Zhang T Zhang W. Metabolites and coronary heart disease: A two sample Mendelian randomization. Int J Cardiol Cardiovasc Risk Prev. (2025) 26:200365. doi: 10.1016/j.ijcrp.2025.200365, PMID: 40487072 PMC12141869

[14] Barbosa-Cortes L Atilano-Miguel S Martin-Trejo JA Jiménez-Aguayo E Martínez-Becerril FI López-Alarcón M. Effect of long-chain omega-3 polyunsaturated fatty acids on cardiometabolic factors in children with acute lymphoblastic leukemia undergoing treatment: a secondary analysis of a randomized controlled trial. Front Endocrinol. (2023) 14:1120364. doi: 10.3389/fendo.2023.1120364, PMID: 37124732 PMC10140550

[15] Li Z-R Wang Y-Y Wang Z-H Qin Q-L Huang C Shi G-S. The positive role of transforming growth factor-β1 in ischemic stroke. Cell Signal. (2024) 121:111301. doi: 10.1016/j.cellsig.2024.111301, PMID: 39019338

[16] Yang Y Li C Yang S Zhang Z Bai X Tang H. Cepharanthine maintains integrity of the blood-brain barrier (BBB) in stroke via the VEGF/VEGFR2/ZO-1 signaling pathway. Aging. (2024) 16:5905–15. doi: 10.18632/aging.205678, PMID: 38517394 PMC11042958

[17] Zhang Q Xiao S Jiao X Shen Y. The triglyceride-glucose index is a predictor for cardiovascular and all-cause mortality in CVD patients with diabetes or pre-diabetes: evidence from NHANES 2001–2018. Cardiovasc Diabetol. (2023) 22:279. doi: 10.1186/s12933-023-02030-z, PMID: 37848879 PMC10583314

[18] Liu D Ren B Tian Y Chang Z Zou T. Association of the TyG index with prognosis in surgical intensive care patients: data from the MIMIC-IV. Cardiovasc Diabetol. (2024) 23:193. doi: 10.1186/s12933-024-02293-0, PMID: 38844938 PMC11157750

[19] Liu C Liang D Xiao K Xie L. Association between the triglyceride-glucose index and all-cause and CVD mortality in the young population with diabetes. Cardiovasc Diabetol. (2024) 23:171. doi: 10.1186/s12933-024-02269-0, PMID: 38755682 PMC11097545

[20] Lertsakulbunlue S Mungthin M Rangsin R Kantiwong A Sakboonyarat B. Trends in baseline triglyceride-glucose index and association with predicted 10-year cardiovascular disease risk among type 2 diabetes patients in Thailand. Sci Rep. (2023) 13:12960. doi: 10.1038/s41598-023-40299-y, PMID: 37563268 PMC10415402

[21] Kong X Gao X Wang W. Oxidative balance score and associations with dyslipidemia and mortality among US adults: A mortality follow-up study of a cross-sectional cohort. J Parenter Enter Nutr. (2024) 48:735–45. doi: 10.1002/jpen.2661, PMID: 38922706

[22] Kwon Y-J Park H-M Lee J-H. Inverse association between oxidative balance score and incident type 2 diabetes mellitus. Nutrients. (2023) 15:2497. doi: 10.3390/nu15112497, PMID: 37299460 PMC10255164

[23] Zhan F Lin G Duan K Huang B Chen L Ni J. Higher oxidative balance score decreases risk of stroke in US adults: evidence from a cross-sectional study. Front Cardiovasc Med. (2023) 10:1264923. doi: 10.3389/fcvm.2023.1264923, PMID: 38034387 PMC10682657

[24] Xu Z Liu D Zhai Y Tang Y Jiang L Li L. Association between the oxidative balance score and all-cause and cardiovascular mortality in patients with diabetes and prediabetes. Redox Biol. (2024) 76:103327. doi: 10.1016/j.redox.2024.103327, PMID: 39186882 PMC11389538

[25] Andres W Rothstein A Elser H Sloane KL Gottesman RF Kasner SE. Trends in the prevalence of stroke among community-dwelling individuals in the US, 1999-2018. JAMA Neurol. (2023) 80:646. doi: 10.1001/jamaneurol.2023.0742, PMID: 37094376 PMC10126939

[26] Zhang W Peng S-F Chen L Chen H-M Cheng X-E Tang Y-H. Association between the oxidative balance score and telomere length from the national health and nutrition examination survey 1999-2002. Oxid Med Cell Longev. (2022) 2022:1–11. doi: 10.1155/2022/1345071, PMID: 35186180 PMC8850082

[27] Guerrero-Romero F Simental-Mendía LE González-Ortiz M Martínez-Abundis E Ramos-Zavala MG Hernández-González SO. The product of triglycerides and glucose, a simple measure of insulin sensitivity. Comparison with the euglycemic-hyperinsulinemic clamp. J Clin Endocrinol Metab. (2010) 95:3347–51. doi: 10.1210/jc.2010-0288, PMID: 20484475

[28] Xu Y Zhuang Y Zhang H. Single and mixed associations of composite antioxidant diet on triglyceride-glucose index. Lipids Health Dis. (2024) 23:254. doi: 10.1186/s12944-024-02233-7, PMID: 39160518 PMC11331692

[29] Van Der Schaft N Schoufour JD Nano J Kiefte-de Jong JC Muka T Sijbrands EJG. Dietary antioxidant capacity and risk of type 2 diabetes mellitus, prediabetes and insulin resistance: the Rotterdam Study. Eur J Epidemiol. (2019) 34:853–61. doi: 10.1007/s10654-019-00548-9, PMID: 31399939 PMC6759671

[30] Lampousi A-M Lundberg T Löfvenborg JE Carlsson S. Vitamins C, E, and β-carotene and risk of type 2 diabetes: A systematic review and meta-analysis. Adv Nutr. (2024) 15:100211. doi: 10.1016/j.advnut.2024.100211, PMID: 38493875 PMC11002795

[31] Cui C Qi Y Song J Shang X Han T Han N. Comparison of triglyceride glucose index and modified triglyceride glucose indices in prediction of cardiovascular diseases in middle aged and older Chinese adults. Cardiovasc Diabetol. (2024) 23:185. doi: 10.1186/s12933-024-02278-z, PMID: 38812015 PMC11138075

[32] Di Marco M Scilletta S Miano N Capuccio S Musmeci M Di Mauro S. Triglycerides to high density lipoprotein cholesterol ratio (TG/HDL), but not triglycerides and glucose product (TyG) index, is associated with arterial stiffness in prediabetes. Diabetes Res Clin Pract. (2025) 224:112189. doi: 10.1016/j.diabres.2025.112189, PMID: 40252776

[33] Von Rauchhaupt E Rodemer C Kliemank E Bulkescher R Campos M Kopf S. Glucose load following prolonged fasting increases oxidative stress- linked response in individuals with diabetic complications. Diabetes Care. (2024) 47:1584–92. doi: 10.2337/dc24-0209, PMID: 38905209 PMC11362116

[34] Butterfield DA Halliwell B. Oxidative stress, dysfunctional glucose metabolism and Alzheimer disease. Nat Rev Neurosci. (2019) 20:148–60. doi: 10.1038/s41583-019-0132-6, PMID: 30737462 PMC9382875

[35] Toyama EQ Herzig S Courchet J Lewis TL Losón OC Hellberg K. Metabolism. AMP-activated protein kinase mediates mitochondrial fission in response to energy stress. Science. (2016) 351:275–81. doi: 10.1126/science.aab4138, PMID: 26816379 PMC4852862

[36] Tangvarasittichai S. Oxidative stress, insulin resistance, dyslipidemia and type 2 diabetes mellitus. World J Diabetes. (2015) 6:456. doi: 10.4239/wjd.v6.i3.456, PMID: 25897356 PMC4398902

[37] Park MH Kim DH Lee EK Kim ND Im DS Lee J. Age-related inflammation and insulin resistance: a review of their intricate interdependency. Arch Pharm Res. (2014) 37:1507–14. doi: 10.1007/s12272-014-0474-6, PMID: 25239110 PMC4246128

[38] de Oliveira Marques S Muller AP Luciano TF Dos Santos Tramontin N da Silva Caetano M Luis da Silva Pieri B. Effects of avocado oil supplementation on insulin sensitivity, cognition, and inflammatory and oxidative stress markers in different tissues of diet-induced obese mice. Nutrients. (2022) 14:2906. doi: 10.3390/nu14142906, PMID: 35889863 PMC9319255

[39] Liu C Liang D. The association between the triglyceride-glucose index and the risk of cardiovascular disease in US population aged ≤ 65 years with prediabetes or diabetes: a population-based study. Cardiovasc Diabetol. (2024) 23:168. doi: 10.1186/s12933-024-02261-8, PMID: 38741118 PMC11092030

[40] Ni C Wang X Zhou Y Wang Q Cai Z Wang H. Association of oxidative balance score, cardiovascular, and all-cause mortality among patients with type 2 diabetes mellitus. Front Endocrinol. (2024) 15:1429662. doi: 10.3389/fendo.2024.1429662, PMID: 39229371 PMC11368781

[41] He G Zhang Z Wang C Wang W Bai X He L. Association of the triglyceride-glucose index with all-cause and cause-specific mortality: a population-based cohort study of 3.5 million adults in China. Lancet Reg Health - West Pac. (2024) 49:101135. doi: 10.1016/j.lanwpc.2024.101135, PMID: 39050982 PMC11263946

[42] Sharif S Groenwold RHH van der Graaf Y Berkelmans GFN Cramer MJ Visseren FLJ. Mediation analysis of the relationship between type 2 diabetes and cardiovascular events and all-cause mortality: Findings from the SMART cohort. Diabetes Obes Metab. (2019) 21:1935–43. doi: 10.1111/dom.13759, PMID: 31062479 PMC6767388

